# A Real-World Comparative Study of Microwave and Radiofrequency Ablation in Treatment-Naïve and Recurrent Hepatocellular Carcinoma

**DOI:** 10.3390/jcm11020302

**Published:** 2022-01-07

**Authors:** Soon Kyu Lee, Dong Jin Chung, Se Hyun Cho

**Affiliations:** 1 Division of Gastroenterology and Hepatology, Department of Internal Medicine, College of Medicine, The Catholic University of Korea, Seoul 06591, Korea; blackiqq@catholic.ac.kr; 2 Department of Radiology, College of Medicine, The Catholic University of Korea, Seoul 06591, Korea; bookdoo7@catholic.ac.kr

**Keywords:** hepatocellular carcinoma, ablation, microwave, radiofrequency, disease-free survival

## Abstract

The efficacy and safety of microwave ablation (MWA) compared to radiofrequency ablation (RFA) for patients with treatment-naïve and recurrent hepatocellular carcinoma (HCC) has not been clarified in Korea. There were 150 HCC patients (100 in the RFA group and 50 in the MWA group) enrolled in our study. The primary outcome was one- and two-year disease-free survival (DFS). Secondary outcomes were complete response (CR) rate, two-year survival rate, risk factors for DFS and complication rate. Treatment outcomes were also assessed using propensity-score matching (PSM). The MWA group had better one- and two-year DFS than the RFA group (*p* = 0.035 and *p* = 0.032, respectively), whereas the CR rate, two-year survival rate, and complication rate were similar between the two groups with fewer major complications in the MWA group (*p* = 0.043). Patients with perivascular tumors, high risk of recurrence, and small tumor size (≤3 cm) were more suitable for MWA than RFA. MWA was also an independent factor for favorable one- and two-year DFS. Finally, the MWA group still showed better one- and two-year DFS than the RFA group after PSM. In conclusion, MWA could be an alternative treatment to RFA especially in patients with a high risk of recurrence, perivascular tumors, and small tumor size.

## 1. Introduction

Hepatocellular carcinoma (HCC) is the fifth most common cancer and the second leading cause of cancer-associated mortality [1,2]. The poor prognosis of HCC is mainly due to late diagnosis at an advanced stage, which prohibits the application of curative therapies [3]. To improve the prognosis of HCC by diagnosing HCC at an early stage, a surveillance strategy using alpha-fetoprotein (AFP) and ultrasonography every six months is recommended for at-risk populations, including those with liver cirrhosis (LC) [4,5]. 

Early detection of small HCC provides the chance for curative treatments such as liver transplantation, liver resection (LR), and radiofrequency ablation (RFA) [6]. However, patients with HCC are still exposed to the risk of recurrence after curative treatments. Indeed, these patients usually have chronic liver diseases including LC, and HCC can recur not only within five years but also more than five years after curative treatments [7]. Therefore, early diagnosis of HCC as well as curative treatments with a low rate of recurrence are the cornerstones for improving the prognosis of patients with HCC.

RFA is the standard of care for early HCC and is comparable with LR in solitary HCCs smaller than 3 cm [8]. RFA treats tumor cells by the generation of an electric current, which causes local heat with a temperature of 60–100 °C that can lead to thermocoagulation necrosis [9]. However, as RFA heats the target area via thermal conduction, the active heating zone around electrodes is only a few millimeters, which causes a decrease in the treatment efficacy for tumors larger than 2–3 cm or those located near a major vessel [10,11]. To complement these limitations, new ablation methods have been developed including microwave ablation (MWA).

MWA, which generates an electromagnetic field, can theoretically reach a higher temperature in a shorter time, create a larger ablation zone, and is less affected by adjacent tissues than RFA [12]. Based on these advantages, MWA has been compared to RFA in several studies, and seems to be an alternative treatment to RFA [13]. However, there has been no real-life comparative study of MWA and RFA in Korea. Moreover, the treatment outcomes of MWA, including patients with recurrent HCC, have not been elucidated.

Herein, we evaluated the treatment outcomes of MWA compared to RFA in Korea for the first time. To reflect real-world data, we also included patients with recurrent HCC and employed propensity-score matching (PSM) analysis. In addition, risk factors for disease-free survival (DFS) and subgroups favoring MWA were also evaluated.

## 2. Materials and Methods

### 2.1. Study Population

In our retrospective cohort study, a total of 261 HCC patients treated with ablation therapy (RFA or MWA) from September 2014 to December 2020 at Yeouido St. Mary’s Hospital (Seoul, Korea) were consecutively enrolled. Of these patients, 111 patients were excluded for the following reasons: ablation therapy for metastatic lesions (*n* = 77), history of both procedures (*n* = 26), intraoperative procedure (*n* = 2), and follow-up of less than three months (*n* = 6). Finally, 150 patients with HCC who were treated with RFA (*n* = 100) or MWA (*n* = 50) were included and analyzed (Supplementary Figure S1). This study was approved by the Institutional Review Board of Yeouido St. Mary’s Hospital (SC21RISI0023), and the requirement to obtain informed consent was waived. The study was conducted in accordance with the Declaration of Helsinki. 

### 2.2. Ablation Therapies

All treatment procedures (RFA and MWA) were performed by an experienced radiologist under monitored anesthesia care. Ultrasound-guided percutaneous ablation was performed during the procedure to monitor treatment. RFA was performed using an RFA system (M-3004; 200-W multifunctional generator; RF Medical, Seoul, Korea) with a 15-G needle electrode. For MWA, an Emprint ablation system (Medtronic, Dublin, UK) with a power of up to 100 W was used. A 13-G straight internally cooled antenna with a frequency of 2450 MHz was applied for MWA therapy. After ablation therapy, patients were monitored for one or two days to observe complications.

### 2.3. Diagnosis and Follow-Up

HCC was diagnosed either by histology or according to the guidelines of the European Association for the Study of the Liver or the American Association for the Study of Liver Disease [4,14,15]. Perivascular HCC was defined based on previous studies using multiphase contrast-enhanced computed tomography (CT) or magnetic resonance imaging (MRI) [16,17]. The tumor stage was classified according to the modified Union for International Cancer Control (mUICC) stage and Barcelona Clinic Liver Cancer (BCLC) stage [15,18].

Technical success was evaluated immediately after ablation therapy by CT, and achievement of complete response (CR) was assessed by CT or MRI two or three months after ablation therapy. Follow-up CT or MRI was performed every three months to evaluate tumor response according to the modified Response Evaluation Criteria in Solid Tumors (mRECIST) [19]. Patients with recurrence after RFA or MWA were treated appropriately according to HCC treatment guidelines [14,15,18].

### 2.4. Definitions and Outcome Measures

Since MWA has been used in recent years of the study, the primary endpoint was one- and two-year DFS, defined as the time interval between the time of RFA or MWA and the time of recurrence, death, or the last follow-up in patients without recurrence, respectively. The second outcomes of the study were two-year overall survival (OS), CR rate, local recurrence rate, and complications. OS was defined as the time interval between the procedure (RFA or MWA) and death or the last follow-up time. The definition of local recurrence was recurrence of the tumor at the adjacent site of treated lesion (≤2.0 cm from its margin) [20]. Patients were monitored for complications after treatment and classified following the definitions of the Society of Interventional Radiology [21]. Major complications included events requiring therapies, prolonged hospitalization, morbidity, or death, with other complications considered as minor. 

### 2.5. Statistical Analysis

Baseline characteristics of patients were expressed as means ± standard deviation for quantitative variables, and as counts (percentage) for categorical variables, as appropriate. Between-group comparisons including the rate of CR, local recurrence, and complications were analyzed by Student’s *t*-test, the chi-squared test, or Fisher’s exact test, as appropriate. Kaplan-Meier analyses were used to demonstrate one- and two-year DFS, and two-year OS according to treatment groups (MWA and RFA group) and subgroups. Since MWA has been used in recent years of the study, we performed log-rank tests truncated at 12 or 24 months of follow-up, as appropriate. To evaluate the risk factors for one- and two-year DFS and two-year OS, significant variables (*p* < 0.05) in univariate analysis were introduced into the multivariate Cox regression method with a backward conditional method. 

Moreover, to reduce selection bias, a one-to-one nearest-neighbor PSM analysis was performed by equating the RFA and MWA groups based on the following variables: sex, age, prior treatment history, cause of HCC, presence of cirrhosis, Child-Turcotte-Pugh (CTP) class, Model for End-stage Liver Disease (MELD) score, tumor size, tumor number, perivascular tumor, BCLC stage, and mUICC stage. *p*-values < 0.05 were considered significant and all statistical analyses were performed using SPSS version 24.0 (IBM, Armonk, NY, USA) and R version 4.0.4 (R Foundation for Statistical Computing, Vienna, Austria).

## 3. Results

### 3.1. Baseline Characteristics

The mean age of participants was 66.0 years and most patients (92.7%) had CTP class A. Of the 150 included patients, 108 (72.0%) were male and 122 (82.0%) had LC. Proportions of patients with a single tumor (73.0% vs. 88.0%, *p* = 0.060) and perivascular tumors (21.0% vs. 34.0%, *p* = 0.127) were not significantly different between the RFA and MWA groups. However, the MWA group (*n* = 50) had more treatment-naïve patients (50% vs. 28%, *p* = 0.013) and larger tumor size (2.1 vs. 1.9 cm, *p* = 0.046) than the RFA group (Table 1).

About half of the patients (54.0% vs. 44.0%) had BCLC stage 0 and the other patients (46.0% vs. 56.0%) had stage A or B without significant differences between the RFA and MWA groups (*p* = 0.326). Most of recurrent HCC patients included in the study had a treatment history of trans-arterial chemoembolization without significant group differences (MWA, 80% vs. RFA, 87.5%; *p* = 0.358). The median follow-up duration of the entire population, the MWA, and the RFA group were 25.3 (Interquartile range [IQR], 12.5–46.1 months), 12.2 (IQR, 9.0–16.1 months), and 39.5 (IQR, 24.9–53.4 months), respectively.

### 3.2. Treatment Outcomes in the Entire Population

**Figure 1:**
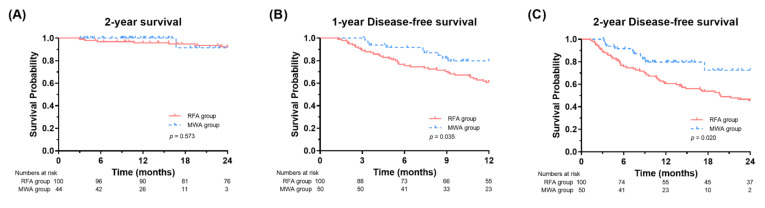
Survival and disease-free survival curves according to the treatment modalities in the entire cohort (**A**–**C**). RFA, radiofrequency ablation; MWA, microwave ablation.

**Figure 2:**
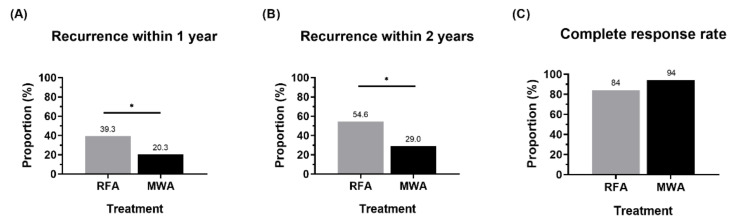
Comparison of recurrent rate (**A**,**B**) and treatment response (**C**) between RFA and MWA. RFA, radiofrequency ablation; MWA, microwave ablation (* *p* < 0.05).

All patients achieved treatment success after MWA or RFA treatment. During the two-year follow-up, eight patients died due to HCC progression (*n* = 7) or hepatic failure (*n* = 1). OS at two years was similar (91.7% vs. 92.4%, *p* = 0.573) between the MWA and RFA groups (Figure 1A). However, the MWA group had significantly better one-year DFS than the RFA group (79.7% vs. 60.7%, *p* = 0.035, respectively) (Figure 1B and Figure 2A). DFS at two years was also better in the MWA group than in the RFA group (72.5% vs. 45.4%, *p* = 0.020, respectively) (Figure 1C and Figure 2B). 

The CR rate was marginally higher in the MWA group than in the RFA group (94.0% vs. 84.0%, *p* = 0.083, respectively) (Figure 2C). Among patients with recurrence, we analyzed the differences in the site of recurrence between the two groups and the proportion of local recurrence were similar between the two groups at one year (*p* = 0.466) and two years (*p* = 0.289) (Supplementary Figure S2). During the two-year follow-up, trans-arterial chemoembolization was the most frequent treatment method (56.9% vs. 80%, respectively) after recurrence, followed by RFA or MWA (39.2% vs. 20.0%, respectively) without significant difference between RFA and MWA groups (Supplementary Table S1).

### 3.3. Subgroup Analysis

**Figure 3:**
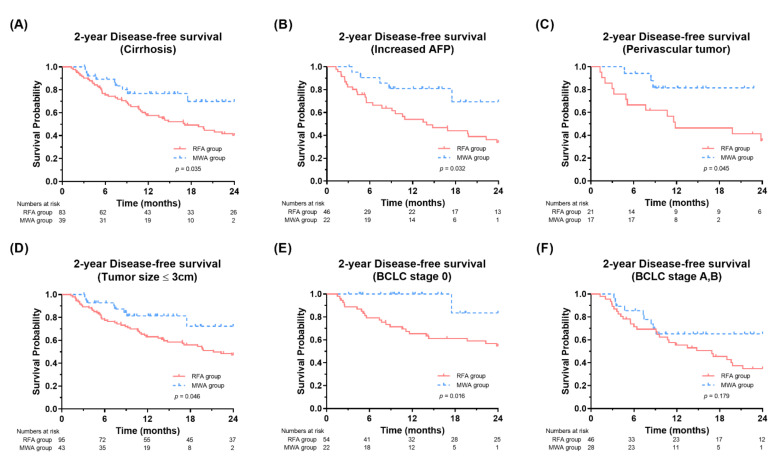
Comparison of disease-free survival in the MWA and RFA groups in subgroups with (**A**) cirrhosis, (**B**) increased AFP, (**C**) perivascular tumor, (**D**) small tumor size (≤3 cm), and (**E**,**F**) BCLC stage 0, A and B. RFA, radiofrequency ablation; MWA, microwave ablation; AFP, alpha-fetoprotein; BCLC, Barcelona Clinic Liver.

We further evaluated the respective outcomes in patients with a high risk of recurrence, including LC and increased AFP levels. In patients with LC and increased AFP levels, the MWA group had a better two-year DFS (*p* = 0.035 and *p* = 0.032, respectively) than the RFA group (Figure 3A,B). When analyzed by tumor site and size, the MWA group achieved significantly better two-year DFS in subgroups with perivascular tumors (*p* = 0.045, Figure 3C) and tumor size ≤3 cm (*p* = 0.046, Figure 3D) than the RFA group. In the analyses according to the BCLC stage, the MWA group exhibited better two-year DFS than the RFA group in patients with BCLC stage 0 (*p* = 0.016, Figure 3E), whereas patients with BCLC stage A or B showed marginally better two-year DFS in the MWA group than in the RFA group (*p* = 0.179, Figure 3F). 

### 3.4. Treatment Complications in the Entire Population

Table 2 shows the summarized data on complications in both groups. Of the 150 included patients, 48 (48.0%) in the RFA group and 19 (38.0%) in the MWA group experienced complications (*p* = 0.246). The MWA group showed a lower rate of major complications than the RFA group (7 of 50 patients [14.0%] vs. 29 of 100 patients [29.0%], *p* = 0.043, respectively) and pain requiring treatment was the most common major complication in both groups (MWA, *n* = 7; RFA, *n* = 27). Fever and pain were the most common minor complications in both groups without significant group differences (*p* = 0.476). All patients who experienced complications (*n* = 67) after ablation therapy were well recovered and tolerable after appropriate management.

### 3.5. Factors Associated with DFS

Of the variables listed in Table 3, treatment-naïve tumors, treatment modality, AFP level, and tumor size were associated with one-year DFS, whereas tumor number, perivascular tumor, and BCLC stage were not significantly associated with one-year DFS. In the multivariate analysis, treatment-naïve tumors (hazard ratio [HR], 0.41; 95% confidence interval [CI], 0.20–0.85; *p* = 0.017), MWA (HR, 0.42; 95% CI, 0.19–0.90; *p* = 0.026), and tumor size (HR, 1.83; 95% CI, 1.29–2.59; *p* = 0.001) were significant factors for one-year DFS.

Treatment-naïve tumors, treatment modality, AFP level, tumor size, and BCLC stage were associated with two-year DFS in the univariate analysis. As with one-year DFS, treatment-naïve tumors (HR, 0.37; 95% CI, 0.19–0.72; *p* = 0.003), MWA (HR, 0.41; 95% CI, 0.20–0.86; *p* = 0.017) and tumor size (HR, 1.78, 95%CI, 1.28–2.47; *p* = 0.001) were independent factors for two-year DFS. However, AFP level and BCLC stage had no significant effect on DFS at two years. 

### 3.6. PSM Analysis 

**Figure 4:**
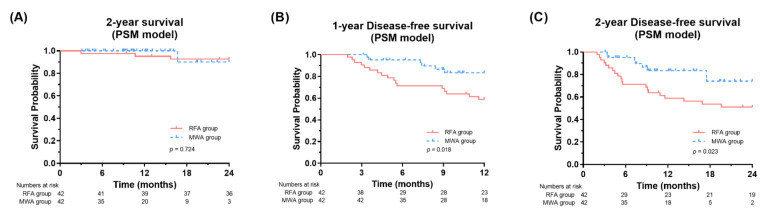
Survival and disease-free survival curves according to treatment modalities in the propensity-score matched cohort (**A**–**C**). RFA, radiofrequency ablation; MWA, microwave ablation.

Finally, we evaluated treatment outcomes according to treatment modalities after PSM. The baseline characteristics, including treatment-naïve tumors and tumor size, were similar between the RFA (*n* = 42) and MWA (*n* = 42) groups after PSM (Table 1). OS at two years was comparable between the two groups (Figure 4A). Meanwhile, DFS at one and two years was still significantly better in the MWA group than in the RFA group (*p* = 0.018 and *p* = 0.023, respectively) (Figure 4B,C).

## 4. Discussion

This is the first study in Korea to compare the outcomes of MWA and RFA therapy for HCC, including patients with recurrent HCC. Our detailed analyses documented better DFS at one and two years with a lower risk of major complications in the MWA group than in the RFA group. Similar trends were observed in subgroups of patients with a high risk of recurrence, perivascular tumor, or small tumor size (≤3 cm). However, the OS at two years and CR rate were similar between the two groups. Thus, these findings suggest that MWA could be applied to patients with treatment-naïve and recurrent HCC with an advantage in treating patients with perivascular tumors, small tumors, and a high risk of recurrence.

In our study, MWA was superior to RFA in terms of one- and two-year DFS before and after PSM and was also a favorable factor for DFS at one and two years. The better DFS of MWA might be attributable to the mechanistic advantages of MWA using electromagnetic fields, which causes a rotation of water and polarization of ions that can lead to a larger active heating zone than RFA, and these results were in accordance with previous studies [12,13,22]. Moreover, this is the first study to document the superior outcomes of MWA including patients with recurrent HCC. Generally, after the recurrence of HCC, the treatment method is selected following the first-line treatment guidelines [15]. Based on our results, MWA might be also applied to patients with recurrent HCC as an alternative to RFA.

Although MWA could generate a larger ablation zone with better DFS than RFA, MWA resulted in similar two-year OS and CR rates to RFA. As most patients (*n* = 147, 98.0%) had small tumors within the Milan criteria, OS at two years was more than 90% in both treatment groups and these high rates of survival might have affected the similar outcomes of both treatments. With longer follow-up, Liu et al. demonstrated that MWA resulted in better five-year OS than RFA in patients within the Milan criteria [22]. Meanwhile, the CR rate of MWA in our study was 94% and these high CR rates of both treatments without significant differences were in line with previous studies [23].

Interestingly, both groups showed similar rates of complications, although MWA was considered to have a higher rate of complications due to a larger ablation zone. Similar to the findings of previous studies, there were no group differences, and the most common complication in both groups was pain with and without treatment [17,23,24]. Moreover, our study demonstrated that the major complication rate in the MWA group was lower than that in the RFA group. This result is different from that of a prior study showing a higher rate of major complications in the MWA group than in the RFA group, which may be due to the fact that the prior study included only patients with treatment-naïve and perivascular tumors [17]. Indeed, our study also included patients with recurrent HCC and our results suggested that MWA was not only effective but also a safe treatment modality for patients with treatment-naïve and recurrent HCC. Further studies with large number of patients are necessary to validate our results about the complications of MWA in patients with treatment-naïve and recurrent HCC.

It remains uncertain which patients are more suitable for MWA than RFA. As RFA is considered susceptible to the “heat-sink” effect and is less effective in perivascular tumors, MWA has been evaluated to be more useful in perivascular tumors. In accordance with several studies, our study also showed better DFS in the MWA group than in the RFA group, and MWA might be considered the technique of choice for perivascular tumors [12,17]. In patients with cirrhosis or an increased level of AFP, which are both known risk factors for HCC [25], MWA also demonstrated better DFS than RFA. Taken together, MWA could be applied as an alternative treatment to RFA for patients at high risk for recurrence and with perivascular tumor.

Moreover, MWA could create a larger ablation zone, and the outcomes of MWA related to tumor size have been studied. According to our study, MWA could be an alternative treatment for patients with an HCC tumor size ≤3 cm, as supported by previous studies [26]. However, most patients (*n* = 138, 92.0%) in our study had a small tumor size (≤3 cm); therefore, further studies are needed to determine the most effective tumor size for MWA. Since MWA can reach its targeted temperature faster, MWA may be beneficial for treating multiple HCCs. However, MWA showed marginally better DFS than RFA in patients with BCLC stage A and B, and including patients with recurrent HCC might contributed to these results due to the increased the possibility of recurrence [7].

Our study has several limitations. First, this study had a retrospective design. This study also had a small number of included patients and a short follow-up duration. Considering small tumor size and early stage of included patients who underwent RFA or MWA, two-year OS might not enough to determine survival differences between RFA and MWA. However, this is the first study to evaluate the efficacy and safety of MWA compared to RFA in Korea. Moreover, for the first time, we included patients with treatment-naïve and recurrent HCC to document the real-life experience of MWA. With detailed analyses including PSM, our study can provide practical information about the outcomes of MWA compared to RFA.

In conclusion, MWA therapy for treatment-naïve and recurrent HCC is safe and provides a better DFS than RFA. Moreover, in patients with a high risk of recurrence, perivascular tumors, or small tumor size, MWA might be a technique of choice and an alternative to RFA with a lower rate of early recurrence. Further large-scale studies are necessary to determine the optimal patient groups for MWA.

## Figures and Tables

**Table 1:** Baseline characteristics of the entire population (general and propensity-score matching model).

Variables	Total (*n* = 150)	General Model			Propensity-Score Matching Model		
		RFA Group (*n* = 100)	MWA Group (*n* = 50)	*p*-Value	RFA Group (*n* = 42)	MWA Group (*n* = 42)	*p*-Value
Male sex (*n*, %)	108 (72.0%)	71 (71.0%)	37 (74.0%)	0.847	30 (71.4%)	32 (76.2%)	0.804
Age, years	66.0 ± 10.2	65.2 ± 9.9	67.7 ± 10.5	0.152	68.4 ± 8.9	66.2 ± 10.4	0.314
Treatment naïve (*n*, %)	53 (35.3%)	28 (28.0%)	25 (50.0%)	0.013	18 (42.9%)	20 (47.6%)	0.826
Cause (*n*, %)				0.822			0.781
Viral/Non-viral	123 (82.0%)/27 (18.0%)	83 (83.0%)/17 (17.0%)	40 (80.0%)/10 (20.0%)		35 (83.3%)/7 (16.7%)	33 (78.6%)/9 (21.4%)	
Cirrhosis	122 (81.3%)	83 (83.0%)	39 (78.0%)	0.604	31 (73.8%)	34 (81.0%)	0.602
CTP class (*n*, %)				1.000			1.000
Class A/class B	139 (92.7%)/11 (7.3%)	93 (93.0%)/7 (7.0%)	46 (92.0%)/4 (8.0%)		41 (97.6%)/1 (2.4%)	40 (95.2%)/2 (4.8%)	
AFP (ng/mL)	63.4 ± 188.7	57.2 ± 161.7	75.9 ± 234.9	0.614	67.6 ± 198.7	66.1 ± 240.3	0.975
MELD score	5.8 ± 2.6	6.0 ± 3.0	5.4 ± 1.7	0.077	5.3 ± 1.2	5.5 ± 1.6	0.516
Tumor size (cm)	2.0 ± 0.8	1.9 ± 0.7	2.1 ± 0.9	0.046	2.0 ± 0.9	2.1 ± 0.8	0.909
Tumor number (*n*, %)				0.060			1.000
Single	117 (78.0%)	73 (73.0%)	44 (88.0%)		37 (88.1%)	37 (88.1%)	
Multiple (2–3)	33 (22.0%)	27 (27.0%)	6 (12.0%)		5 (11.9%)	5 (11.9%)	
Perivascular tumor (*n*, %)	38 (25.3%)	21 (21.0%)	17 (34.0%)	0.127	12 (28.6%)	12 (28.6%)	1.000
BCLC stage (*n*, %)				0.326			0.662
Stage 0	76 (50.7%)	54 (54.0%)	22 (44.0%)		22 (52.4%)	19 (45.2%)	
Stage A, B	74 (49.3%)	46 (46.0%)	28 (56.0%)		20 (47.6%)	23 (54.8%)	
mUICC stage (*n*, %)				0.326			0.662
Stage I	76 (50.7%)	54 (54.0%)	22 (44.0%)		22 (52.4%)	19 (45.2%)	
Stage II, III	74 (47.3%)	46 (46.0%)	28 (56.0%)		20 (47.6%)	23 (54.8%)	

RFA, radiofrequency ablation; MWA, microwave ablation; CTP, Child-Turcotte-Pugh; MELD, model for end-stage liver disease; AFP, al-pha-fetoprotein; IQR, interquartile range; BCLC, Barcelona Clinic Liver Cancer; mUICC, modified Union for International Cancer Control.

**Table 2:** Comparison of treatment complications between RFA and MWA in the entire population.

Complications	RFA Group (*n* = 100)	MWA Group (*n* = 50)	*p*-Value
All complications	48 (48.0%)	19 (38.0%)	0.246
Major complications	29 (29.0%)	7 (14.0%)	0.043
pain requiring treatment	27	7	
Hydrothorax with drain	1	0	
Hemoperitoneum	1	0	
Minor complications	19 (19.0%)	12 (24.0%)	0.476
pain without treatment	10	8	
Fever without infection	6	3	
Nausea and vomiting	3	1	

RFA, radiofrequency ablation; MWA, microwave ablation.

**Table 3:** Univariate and multivariate Cox regression analysis for disease-free survival.

Variables	One-Year Disease-Free Survival				Two-Year Disease-Free Survival			
	Univariate Analysis		Multivariate Analysis		Univariate Analysis		Multivariate Analysis	
	HR (95% CI)	*p*-Value	HR (95% CI)	*p*-Value	HR (95% CI)	*p*-Value	HR (95% CI)	*p*-Value
Age (years)	0.98 (0.96–1.01)	0.223			0.99 (0.96–1.01)	0.295		
Male sex	1.34 (0.68–2.64)	0.393			1.14 (0.64–2.01)	0.663		
Treatment naïve	0.35 (0.17–0.72)	0.004	0.41 (0.20–0.85)	0.017	0.32 (0.17–0.62)	0.001	0.37 (0.19–0.72)	0.003
Cause of HCC								
Viral vs. Non-viral	0.90 (0.42–1.93)	0.786			0.81 (0.41–1.60)	0.549		
Cirrhosis	2.00 (0.79–5.06)	0.142			2.31 (0.99–5.36)	0.052		
Treatment modality								
MWA vs. RFA	0.47 (0.23–0.96)	0.040	0.42 (0.19–0.90)	0.026	0.45 (0.23–0.90)	0.024	0.41 (0.20–0.86)	0.017
CTP class B vs. class A	1.34 (0.48–3.72)	0.581			1.06 (0.38–2.91)	0.916		
AFP (ng/mL)	1.00 (1.00–1.00)	0.019	1.00 (1.00–1.00)	0.246	1.00 (1.00–1.00)	0.035	1.00 (1.00–1.00)	0.280
MELD score	0.99 (0.89–1.11)	0.847			0.96 (0.86–1.08)	0.514		
Tumor size (cm)	1.62 (1.15–2.28)	0.005	1.83 (1.29–2.59)	0.001	1.53 (1.12–2.10)	0.007	1.78 (1.28–2.47)	0.001
Tumor number								
Multiple vs. single	1.38 (0.73–2.62)	0.321			1.69 (0.98–2.91)	0.057		
Perivascular tumor	1.28 (0.68–2.38)	0.446			1.13 (0.64–2.00)	0.672		
BCLC stage								
Stage A,B vs. stage 0	1.76 (0.98–3.16)	0.061			1.80 (1.07–3.01)	0.026	1.15 (0.57–2.31)	0.694

HR, hazard ratio; CI, confidence interval; HCC, hepatocellular carcinoma; MWA, microwave ablation; RFA, radiofrequency ablation; CTP, Child-Turcotte-Pugh; AFP, alpha-fetoprotein; MELD, model for end-stage liver disease; BCLC, Barcelona clinic liver cancer.

## Data Availability

Data are not available due to ethical issues.

## Supplementary Materials

The following supporting information can be downloaded at: www.mdpi.com/article/10.3390/jcm11020302/s1. Figure S1: Study flow chart; Figure S2: The comparison of recurrent site between the MWA and RFA group among patients with recurrence; Table S1: Treatment modalities after recurrence in recurrent patients of the RFA and MWA groups for two years follow-up.
