# Supplementary material for: A Real-World Comparative Study of Microwave and Radiofrequency Ablation in Treatment-Naïve and Recurrent Hepatocellular Carcinoma
Source: J Clin Med. 2022 Jan 7;11(2):302. doi: 10.3390/jcm11020302 (PMC8778377; doi:10.3390/jcm11020302)
Supplement: Supplementary file 1 [file jcm-11-00302-s001.zip › jcm-1445055-supplementary.pdf]

**Supplementary Materials**

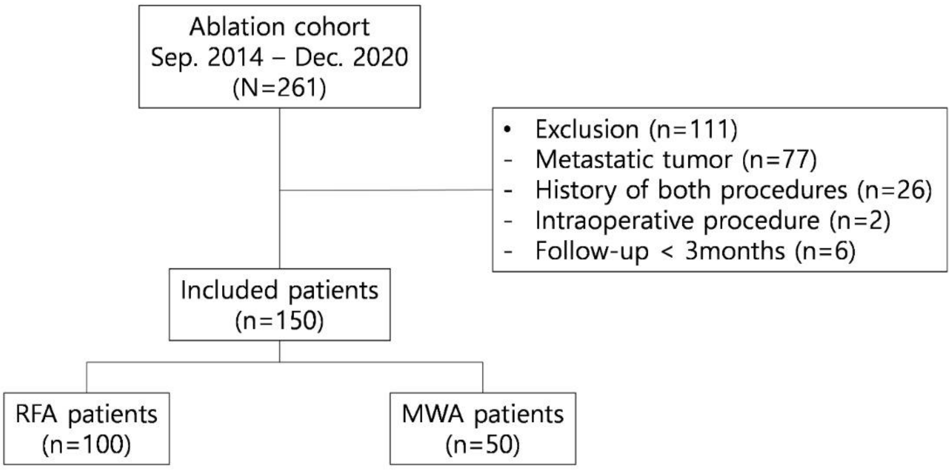


**Figure S1.** Study flow chart. RFA, radiofrequency ablation; MWA, microwave ablation.

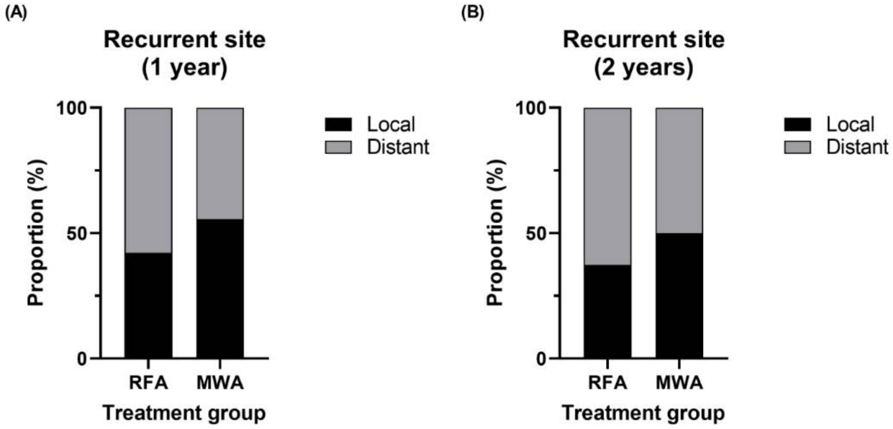


**Figure S2.** The comparison of recurrent site between the MWA and RFA group among patients with recurrence.

**Table S1.** Treatment modalities after recurrence in recurrent patients of the RFA and MWA groups for 2 years follow-up.

| Recurrent patients | RFA group (n=51) | MWA group (n=10) | *p*-Value |
|---|---|---|---|
| Treatment modalities | | | 0.370 |
| TACE | 29 (56.9%) | 8 (80.0%) | |
| RFA or MWA | 20 (39.2%) | 2 (20.0%) | |
| Other treatments | 2 (3.9%) | 0 (0.0%) | |

RFA, radiofrequency ablation; MWA, microwave ablation; TACE, trans-arterial chemoembolization.
